# Are Dominant Strictures in Primary Sclerosing Cholangitis a Risk Factor for Cholangiocarcinoma?

**DOI:** 10.1007/s11901-017-0341-2

**Published:** 2017-04-27

**Authors:** Roger W Chapman, Kate D. Williamson

**Affiliations:** 1 Nuffield Department of Medicine, Oxford University, Oxford, UK; 2 Translational Gastroenterology Unit, Oxford University Hospital, Oxford, UK; 3 Translational Gastroenterology Unit, John Radcliffe Hospital, Headley Way, Headington, Oxford, OX3 9DU UK

**Keywords:** Primary sclerosing cholangitis, Cholangiocarcinoma, Dominant stricture, Inflammatory bowel disease, Cholangioscopy

## Abstract

**Purpose of Review:**

Cholangiocarcinoma is a devastating, unpredictable complication of large duct primary sclerosing cholangitis (PSC), which occurs in 5–15% of patients. The aim of this review is to discuss whether dominant strictures (DS) occurring in the larger bile ducts in PSC are a risk factor for the development of cholangiocarcinoma.

**Recent Findings:**

The development of DS is related to specific genetic polymorphisms affecting the innate immune system and the microbiome. In a recent study, the mean survival of PSC patients with DS was much worse (13.7 years) than for those without a DS (23 years). Survival difference was related to a 26% risk of cholangiocarcinoma, which developed only in those with DS. Half of the patients with cholangiocarcinoma presented within 4 months of the diagnosis of PSC. In another study, the risk of developing cholangiocarcinoma was directly related to the presence of underlying IBD, although this remains controversial. Efforts are being made towards surveying for cholangiocarcinoma including magnetic resonance imaging, endoscopic surveillance and serum tumour markers, but so far, an effective surveillance strategy has not been identified. DS should be treated endoscopically in the setting of symptoms, and there is limited evidence to suggest this may impact protectively on progression to cholangiocarcinoma.

**Summary:**

It is established that the presence of symptomatic DS occurring in the larger bile ducts in PSC can be the first presentation of cholangiocarcinoma. There is an increasing body of evidence that even when proven to be benign, dominant biliary strictures predispose to the future development of cholangiocarcinoma. Regular surveillance should be targeted at this selected high-risk group of PSC patients.

## Introduction

Primary sclerosing cholangitis (PSC) is a rare, progressive, cholestatic liver disease characterised by inflammation, stricturing and obliterative fibrosis of the biliary system, ultimately leading to biliary cirrhosis, portal hypertension and eventually hepatic failure in the majority of patients [1]. The clinical course of large duct PSC is highly variable and unpredictable [1, 2]. Whilst the median survival from presentation to death or liver transplantation in symptomatic patients is approximately 10 to 12 years, 75% of asymptomatic patients will survive 15 years or more. A recent Dutch study has shown an overall median survival of 22 years in an unselected population of PSC patients [3].

In the past, the majority of patients with PSC died of hepatic failure following deepening, cholestatic jaundice. However, over the last 20 years with the advent of successful transplantation, the majority (40–50%) of patients with large duct PSC die of malignancy, either hepatobiliary (cholangiocarcinoma, gall bladder cancer or hepatoma in cirrhotic patients) or colonic adenocarcinoma in PSC patients with associated inflammatory bowel disease (IBD) [4]. The reason for the high malignancy rate is probably explained by chronic inflammation in the biliary system and the colon, although whether PSC patients have a particular genetic susceptibility to develop cancer is unclear.

It is apparent that, despite the strong association with non-smoking, large duct PSC is a premalignant disease. In contrast, small duct PSC has not been associated with an increased risk of either biliary or colonic neoplasia and has a much better prognosis [5].

## Hepatobiliary Cancer

As stated above, approximately 5 to 15% of patients with large duct PSC die from the development of bile duct carcinoma, which often follows a very aggressive course [6–8]. Approximately 0.5 to 1.0% of patients with large duct PSC will develop cholangiocarcinoma (CCA) or gall bladder cancer every year [8].

Unfortunately, at present, there are no factors that will predict which patients will develop this cancer. Tumour markers such as carcinogenic embryonic antigen (CEA) and carbohydrate antigen 19-9 (CA 19-9) have been investigated as potential serum markers of the development of bile duct cancer in PSC. Although some centres have found elevations in serum CA 19-9 a useful predictor, these results have not been confirmed in other units [8].

The median survival after the diagnosis of cholangiocarcinoma in PSC is only 9–12 months, although liver transplantation after chemotherapy is possible in selected patients with small tumours (<3.0 cm) and no evidence of metastatic disease [3, 9]. In this highly selected group, the survival is comparable with PSC without cholangiocarcinoma, viz. a 65–80% 5-year survival post transplantation [10, 11]. In PSC patients who are found to have unsuspected bile duct cancers at the time of liver transplantation, there is an approximately 35% 5-year survival [12]. Thus, there is clearly an unmet need to identify subgroups of PSC patients at particular high risk of developing cholangiocarcinoma, where heightened surveillance may be helpful in identifying cholangiocarcinoma at an early treatable stage.

## Dominant Strictures in PSC

**Fig. 1:**
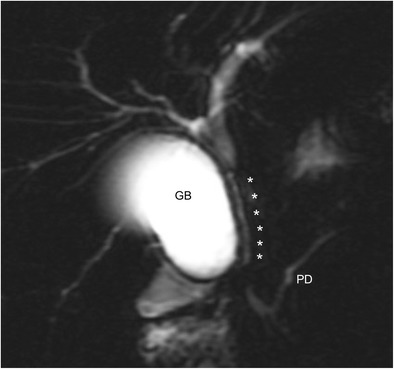
Magnetic resonance cholangiopancreatography (MRCP) showing a dominant stricture. This is a thick-slab heavily T2-weighted MRCP image which shows a long dominant stricture involving the entire length of the common bile duct and the distal common hepatic duct. The dominant stricture is indicated by the *six asterisks* to its *right*. The proximal common hepatic duct just above it and intrahepatic ducts have resultant dilatation. *GB* gall bladder, *PD* pancreatic duct. Figure courtesy of Dr Helen K. Bungay, John Radcliffe Hospital, Oxford, UK

Patients with large duct PSC may develop progressive jaundice, worsening of their liver biochemistry, or symptoms of cholangitis, prompting investigation for a dominant stricture (DS). A DS in PSC is defined cholangiographically as a stricture less than 1.5-mm diameter in the common bile duct, or less than 1 mm in the left or right main hepatic ducts [13]. (See Fig. 1.)

Approximately 10–62% of patients with PSC will develop a DS at some stage in their disease [14••, 15]. The development of dominant bile strictures in PSC has been associated with CD14 receptor signalling. CD14 is known to be a key mediator of the innate immune system, and its common genetic variant has been associated with the development of both alcoholic liver disease and non-alcoholic steatohepatitis [16, 17]. The variant CD14-260C>T polymorphism was associated with the development of DS and an increased risk of cholangitis. It appears from this data that the innate immune response may be important during biliary stricture formation [18].

The importance of genetic factors in the development of DS in PSC was further emphasised by a recent genome-wide association study which identified the FUT2 secretor status and genotype, the single-nucleotide polymorphism rs601338, as a potential genetic risk factor in PSC, which significantly influences biliary bacterial composition [19]. Presence of this genotype in PSC has been strongly associated with episodes of cholangitis, fungobilia and the incidence of dominant stenosis [20•].

## The Effect of Dominant Stricture on Prognosis and the Risk of Cholangiocarcinoma

The presence of a DS has been associated with a worsened long-term prognosis. This was demonstrated in a 25-year study of 128 PSC patients from a tertiary referral centre in the UK [14••]. In this selected population, the mean survival of those with DS was much worse (13.7 years) than for those without a DS (23 years). It is noteworthy that much of the survival difference was related to a 26% risk of cholangiocarcinoma, which developed only in those with DS. Half of the patients with cholangiocarcinoma presented within 4 months of the diagnosis of PSC, emphasising the importance of thorough evaluation of new DS.

These findings have been confirmed from a German study, namely that the presence of DS in PSC is associated with a worse prognosis and an increased risk of carcinomas both in the bile duct and colon [21]. Furthermore, this study suggested that the risk of developing cholangiocarcinoma (and colonic cancer) was directly related to the presence of underlying IBD [22••]. In this study, a group of 171 patients with PSC were followed prospectively for as long as 20 years; 20 had DS at entry and a further 77 patients developed a dominant stenosis over the course of follow-up. These patients were treated endoscopically by repeated balloon dilatations, and five patients were temporarily stented. In patients with DS without IBD, no cholangiocarcinoma occurred and the actuarial survival free of transplantation was 77.8% at 18 years. In contrast, the 18-year survival was only 23% in the PSC with a DS and IBD, and six patients in this group developed a cholangiocarcinoma and two patients gall bladder over the 20-year follow-up. The presence of IBD had no impact on survival in those without a DS. In the DS group, bacteria in the bile had no effect on survival whereas *Candida* in the bile was associated with reduced survival.

The findings relating to the effect of IBD on the natural history of DS were not confirmed in a cohort of 241 Dutch PSC patients followed for a mean of 6 years [23]. The main difference was that 11 malignancies (seven colorectal cancer and four biliary malignancies) were observed in the group without DS (irrespective of IBD status). Overall, the cancer risk in their cohort appeared to be more dependant on the presence of a DS rather than the combination of concurrent IBD and a dominant biliary stricture.

Taken together, the limited data suggest that the presence of a DS, regardless of whether it occurs at the diagnosis of PSC or develops during the course of follow-up, represents an increased risk of both cholangiocarcinoma and reduced survival. A summary of these studies pertaining to the risk of DS leading to cholangiocarcinoma is presented in Table 1.

**Table 1:** Studies which address the risk of the presence of a dominant stricture on development of cholangiocarcinoma

Study	Country	Length of FU (mean)	Number of patients studied	Presence of DS, *n* (%)	Diagnosis of CCA during FU		Comments
					DS *n* (%)	No DS, *n* (%)	
Rudolph G. et al., 2009 [21]	Germany	6.9 years	171	97 (56.7)	6 (6.2)	0 (0)	Prospective study Association of IBD as well as DS with development of CCA
Janse M. et al., 2012 [23]	Netherlands	6.2 years	241	77 (31.9)	9 (11.7)	2 (2.4)	Retrospective study No association with IBD Published in letter to editor style only
Chapman M. et al., 2009 [14••]	UK	9.8 years	128	80 (62.5)	21 (26.3)	0 (0)	Retrospective study 48% of CCA were within 4 months of PSC diagnosis

There are several other studies that examine risk of dominant stricture on overall survival free of liver transplantation, but few studies which specifically address the risk of developing cholangiocarcinoma

*Abbreviations*: *CCA* cholangiocarcinoma, *DS* dominant stricture, *FU* follow-up, *IBD* inflammatory bowel disease, *PSC* primary sclerosing cholangitis

## Dilatation of Dominant Strictures and Possible Reduction of Cancer Risk

If symptomatic, dominant strictures should be dilated with endoscopic balloon dilatation via endoscopic retrograde cholangiopancreatography (ERCP) and may often require multiple dilatations over time. Occasionally, temporary stent insertion is required. Current evidence is conflicting as to whether temporary stenting after balloon dilatation offers any extra benefit, and a randomised multicentre European trial is in progress to address this issue [24].

Antibiotics, such as quinolones, should be administered in the setting of acute cholangitis related to a DS, and prophylactic antibiotics, such as low-dose ciprofloxacin, are sometimes required in the clinical setting of recurrent cholangitis. As discussed previously, a DS may be benign or represent the presentation of cholangiocarcinoma, the latter being notoriously difficult to diagnose. The explanation for the high rate of malignancy is probably related, in part, to chronic inflammation secondary to biliary obstruction, as cholangiocarcinoma also develops in patients with secondary sclerosing cholangitis. It follows, therefore, that effective dilatation of dominant strictures relieving the obstruction to bile flow should reduce chronic inflammation and the cancer risk.

Some support for this hypothesis comes from a long-term study from Germany, already described above [15, 22••]. From the group of 171 patients followed over 20 years, only 8 patients developed a biliary tract cancer, which is below the predicted level. However, the study was uncontrolled, and further studies are required to confirm this.

## Surveillance for Development of Cholangiocarcinoma in Dominant Strictures in PSC

Surveillance strategies in PSC patients for the detection of cholangiocarcinoma at an early, potentially treatable stage have failed to date. Serum tumour markers such as CEA and CA 19-9 have been investigated as potential serum markers of the development of bile duct cancer in PSC but have a low sensitivity and specificity. Unfortunately, by the time the serum CA 19-9 is significantly persistently elevated, the carcinoma is usually too advanced for curable treatment [25]. Moreover, approximately 7% of the population are unable to express CA 19-9 due to genetic variation in the *FUT3* gene [26, 27••].

Annual magnetic resonance cholangiography (MRCP) surveillance for cholangiocarcinoma in PSC is widely employed although the technique has not yet proven to be of benefit [8]. Advances in MRCP technology may offer greater sensitivity and specificity than currently obtained with 3D MRCP.

It has been suggested in a recent Finnish study that PSC patients with advanced bile duct disease, which included DS patients, should receive regular surveillance by ERCP and brush cytology every 6 months to 2 years [28]. PSC patients with suspicious changes on ploidy analysis with DNA flow cytometry were referred for liver transplantation to be performed at an early treatable stage. This aggressive approach has not been adopted by many units, as in practice the exclusion of possible cholangiocarcinoma in PSC patients with a DS is often difficult. Despite having a high specificity, brush cytology of biliary strictures performed at ERCP is often non-confirmatory, with unacceptably low levels of sensitivity obtained in most centres. A recent meta-analysis showed a pooled sensitivity of only 43%, making it a poor tool for diagnosis of cholangiocarcinoma [29]. In addition, one must bear in mind the risks posed in employing ERCP in the patient with PSC, not least including the risk of introducing bacteria and the possibility of invoking (recurrent) cholangitis [30].

Fluorescence in situ hybridisation (FISH) analysis has been advocated as increasing the sensitivity and specificity of cytological analysis. Chromosomal polysomy detected by FISH has been shown to identify patients with early cholangiocarcinoma or those patients with a high risk of developing cholangiocarcinoma. Up to 80% of biliary malignancies show abnormalities in chromosomal number (i.e. aneuploidy) and/or structure, which can be detected by FISH [31]. It has recently been demonstrated that patients with serial biliary polysomy have high risk of developing cholangiocarcinoma, although patients with reversion of polysomy (greater than 50% of the PSC patients studied) have a decreased risk [32•].

Per-oral video cholangioscopy has been studied as a mode of potentially increasing the sensitivity of diagnosis of cholangiocarcinoma in the setting of DS in PSC. In a prospective study from the Mayo Clinic of 30 patients undergoing ERCP for DS, 4 patients ultimately had cholangiocarcinoma, but the use of cholangioscopy did not improve detection beyond routine ERCP [33]. Other small studies have postulated a potential minor improvement of cholangioscopy on detecting cholangiocarcinoma beyond ERCP [34, 35]. Annual cholangioscopy with biliary biopsies, analogous to yearly colonoscopy for the early detection of colonic carcinoma in PSC, is being studied prospectively in high-risk PSC patients with extrahepatic disease, particularly those patients with DS.

## Conclusion

There is an unmet need to identify subgroups of PSC patients at particular high risk of developing cholangiocarcinoma, where heightened surveillance may be helpful in identifying cholangiocarcinoma at an early treatable stage. There is an increasing body of evidence that even when the stricture(s) is proven to be benign, dominant biliary strictures predispose to the future development of cholangiocarcinoma in patients with PSC. Additionally, PSC patients with associated IBD may be at particularly high risk. Currently, there is no proven beneficial form of surveillance strategy for cholangiocarcinoma, and studies are ongoing to address this issue.

## References

[1] Hirschfield GM, Karlsen TH, Lindor KD, Adams DH (2013). Primary sclerosing cholangitis. Lancet.

[2] Karlsen TH, Boberg KM (2013). Update on primary sclerosing cholangitis. J Hepatol.

[3] Boonstra K, Weersma RK, van Erpecum KJ, Rauws EA, Spanier BW, Poen AC (2013). Population-based epidemiology, malignancy risk, and outcome of primary sclerosing cholangitis. Hepatology.

[4] Bergquist A, Ekbom A, Olsson R, Kornfeldt D, Loof L, Danielsson A (2002). Hepatic and extrahepatic malignancies in primary sclerosing cholangitis. J Hepatol.

[5] Bjornsson E, Olsson R, Bergquist A, Lindgren S, Braden B, Chapman RW (2008). The natural history of small-duct primary sclerosing cholangitis. Gastroenterology.

[6] Fevery J, Verslype C, Lai G, Aerts R, Van Steenbergen W (2007). Incidence, diagnosis, and therapy of cholangiocarcinoma in patients with primary sclerosing cholangitis. Digestive Diseases & Sciences.

[7] Claessen MMH, Vleggaar FP, Tytgat KMAJ, Siersema PD, van Buuren HR. High lifetime risk of cancer in primary sclerosing cholangitis. J Hepatol. 50(1):158–64. doi:10.1016/j.jhep.2008.08.013. 10.1016/j.jhep.2008.08.013 19012991

[8] Razumilava N, Gores GJ, Lindor KD (2011). Cancer surveillance in patients with primary sclerosing cholangitis. Hepatology.

[9] Williamson KD, Chapman RW (2015). Primary sclerosing cholangitis: a clinical update. Br Med Bull.

[10] Darwish Murad S, Kim WR, Harnois DM, Douglas DD, Burton J, Kulik LM (2012). Efficacy of neoadjuvant chemoradiation, followed by liver transplantation, for perihilar cholangiocarcinoma at 12 US centers. Gastroenterology.

[11] Rosen CB, Heimbach JK, Gores GJ (2010). Liver transplantation for cholangiocarcinoma. Transpl Int.

[12] Brandsaeter B, Isoniemi H, Broome U, Olausson M, Backman L, Hansen B (2004). Liver transplantation for primary sclerosing cholangitis; predictors and consequences of hepatobiliary malignancy. J Hepatol.

[13] European Association for the Study of the L (2009). EASL Clinical Practice Guidelines: management of cholestatic liver diseases. J Hepatol.

[14] Chapman MH, Webster GJM, Bannoo S, Johnson GJ, Wittmann J, Pereira SP (2012). Cholangiocarcinoma and dominant strictures in patients with primary sclerosing cholangitis: a 25-year single-centre experience. Eur J Gastroenterol Hepatol.

[15] Gotthardt DN, Rudolph G, Klöters-Plachky P, Kulaksiz H, Stiehl A (2010). Endoscopic dilation of dominant stenoses in primary sclerosing cholangitis: outcome after long-term treatment. Gastrointest Endosc.

[16] Järveläinen HA, Orpana A, Perola M, Savolainen VT, Karhunen PJ, Lindros KO (2001). Promoter polymorphism of the CD14 endotoxin receptor gene as a risk factor for alcoholic liver disease. Hepatology.

[17] Brun P, Castagliuolo I, Floreani AR, Buda A, Blasone L, Palù G (2006). Increased risk of NASH in patients carrying the C(−159)T polymorphism in the CD14 gene promoter region. Gut.

[18] Friedrich K, Smit M, Brune M, Giese T, Rupp C, Wannhoff A (2016). CD14 is associated with biliary stricture formation. Hepatology.

[19] Folseraas T, Melum E, Rausch P, Juran BD, Ellinghaus E, Shiryaev A (2012). Extended analysis of a genome-wide association study in primary sclerosing cholangitis detects multiple novel risk loci. J Hepatol.

[20] • Rupp C, Friedrich K, Folseraas T, Wannhoff A, Bode KA, Weiss KH, et al. Fut2 genotype is a risk factor for dominant stenosis and biliary candida infections in primary sclerosing cholangitis. Aliment Pharmacol Ther. 2014;39(8):873–82. doi:10.1111/apt.12663. **This is an important study showing that genetic factors can affect the biome and biliary infection with the development of cholangiocarcinoma**. 10.1111/apt.12663 24612312

[21] Rudolph G, Gotthardt D, Kloters-Plachky P, Kulaksiz H, Rost D, Stiehl A (2009). Influence of dominant bile duct stenoses and biliary infections on outcome in primary sclerosing cholangitis. J Hepatol.

[22] Rudolph G, Gotthardt D, Kloeters-Plachky P, Rost D, Kulaksiz H, Stiehl A (2010). In PSC with dominant bile duct stenosis, IBD is associated with an increase of carcinomas and reduced survival. J Hepatol.

[23] Janse M, Lamberts LE, Verdonk RC, Weersma RK. IBD is associated with an increase in carcinoma in PSC irrespective of the presence of dominant bile duct stenosis. Journal of hepatology. 57(2):473–4. doi:10.1016/j.jhep.2012.02.034. 10.1016/j.jhep.2012.02.034 22537688

[24] Clinical Trials identifier: NCT01398917. Short-term stenting versus balloon dilatation for dominant strictures in primary sclerosing cholangitis. URL: https://clinicaltrials.gov/ct2/show/NCT01398917. Accessed 22/02/2017.

[25] Chapman R, Fevery J, Kalloo A, Nagorney DM, Boberg KM, Shneider B (2010). Diagnosis and management of primary sclerosing cholangitis. Hepatology.

[26] Nishihara S, Narimatsu H, Iwasaki H, Yazawa S, Akamatsu S, Ando T (1994). Molecular genetic analysis of the human Lewis histo-blood group system. J Biol Chem.

[27] Rizvi S, Eaton JE, Gores GJ (2015). Primary sclerosing cholangitis as a premalignant biliary tract disease: surveillance and management. Clin Gastroenterol Hepatol.

[28] Boyd S, Mustonen H, Tenca A, Jokelainen K, Arola J, Färkkilä MA (2017). Surveillance of primary sclerosing cholangitis with ERC and brush cytology: risk factors for cholangiocarcinoma. Scand J Gastroenterol.

[29] Trikudanathan G, Navaneethan U, Njei B, Vargo JJ, Parsi MA (2014). Diagnostic yield of bile duct brushings for cholangiocarcinoma in primary sclerosing cholangitis: a systematic review and meta-analysis. Gastrointest Endosc.

[30] Van den Hazel S, Wolfhagen F, Van Buuren H, van de Meeberg P, Van Leeuwen D (2000). Prospective risk assessment of endoscopic retrograde cholangiography in patients with primary sclerosing cholangitis. Dutch PSC Study Group. Endoscopy.

[31] Bangarulingam SY, Bjornsson E, Enders F, Barr Fritcher EG, Gores G, Halling KC (2010). Long-term outcomes of positive fluorescence in situ hybridization tests in primary sclerosing cholangitis. Hepatology.

[32] Quinn KP, Tabibian JH, Lindor KD (2017). Clinical implications of serial versus isolated biliary fluorescence in situ hybridization (FISH) polysomy in primary sclerosing cholangitis. Scand J Gastroenterol.

[33] Azeem N, Gostout CJ, Knipschield M, Baron TH (2014). Cholangioscopy with narrow-band imaging in patients with primary sclerosing cholangitis undergoing ERCP. Gastrointest Endosc.

[34] Arnelo U, von Seth E, Bergquist A (2015). Prospective evaluation of the clinical utility of single-operator peroral cholangioscopy in patients with primary sclerosing cholangitis. Endoscopy.

[35] Tischendorf JJ, Kruger M, Trautwein C, Duckstein N, Schneider A, Manns MP (2006). Cholangioscopic characterization of dominant bile duct stenoses in patients with primary sclerosing cholangitis. Endoscopy.

